# Development of a CRISPR/Cas12a-based fluorescent detection method of Senecavirus A

**DOI:** 10.1186/s12917-024-04116-6

**Published:** 2024-06-14

**Authors:** Wei He, Kai Liao, Ruixue Li, Wanqing Peng, Bingxu Qian, Dexin Zeng, Fang Tang, Feng Xue, Yong Sam Jung, Jianjun Dai

**Affiliations:** 1 MOE Joint International Research Laboratory of Animal Health and Food Safety, Nanjing Agricultural University, Nanjing, 210095 China; 2 Ningxia Hui Autonomous Region Food Testing and Research Institute, Yinchuan, 750002 China; 3 Sanya Institute of Nanjing Agricultural University, Sanya, 572024 China; 4 China Pharmaceutical University, Nanjing, 211198 China

**Keywords:** PIVD, Senecavirus A, CRISPR/Cas12a, Ultra-sensitivity, Rapid diagnosis

## Abstract

**Background:**

Senecavirus A (SVA), identified in 2002, is known to cause porcine idiopathic vesicular disease (PIVD), which presents with symptoms resembling other vesicular diseases. This similarity complicates field diagnosis. Conventional molecular diagnostic techniques are limited by their cost, sensitivity, and requirement for complicated instrumentation. Therefore, developing an effective and accurate diagnostic method is crucial for timely identification and isolation of affected pigs, thereby preventing further disease spread.

**Methods:**

In this study, we developed a highly-specific and ultra-sensitive SVA detection method powered by CRISPR/Cas12a. To enhance the availability in laboratories with varied equipment conditions, microplate reader and ultraviolet light transilluminator were introduced. Moreover, PCR amplification has also been incorporated into this method to improve sensitivity. The specificity and sensitivity of this method were determined following the preparation of the recombinant Cas12a protein and optimization of the CRISPR/Cas12a-based trans-cleavage system.

**Results:**

The method demonstrated no cross-reactivity with ten kinds of viruses of swine. The minimum template concentration required to activate substantial trans-cleavage activity was determined to be 10^6^ copies/µL of SVA templates. However, when PCR amplification was incorporated, the method achieved a detection limit of one copy of SVA templates per reaction. It also exhibited 100% accuracy in simulated sample testing. The complete testing process does not exceed three hours.

**Conclusions:**

Importantly, this method utilizes standard laboratory equipment, making it accessible for use in resource-limited settings and facilitating widespread and ultra-sensitive screening during epidemics. Overall, the development of this method not only broadens the array of tools available for detecting SVA but also holds significant promise for controlling the spread of PIVD.

**Supplementary Information:**

The online version contains supplementary material available at 10.1186/s12917-024-04116-6.

## Background

Senecavirus A (SVA), also referred to as Seneca Valley virus, is the sole species of the *Senecavirus* genus within the *Picornaviridae* family [1]. Initially identified in 2002, SVA was discovered by chance during the cultivation of adenovirus-5-based vectors in PER.C6 cells [2]. Early research highlighted its notable oncolytic properties against human tumor cells [3, 4]. In 2007, SVA was implicated as the causative agent of porcine idiopathic vesicular disease (PIVD) [5]. It is a non-enveloped, positive-sense, single-stranded RNA virus, composed of four structural and seven non-structural proteins [6]. Since the initial outbreak in the United States [7], subsequent outbreaks in Thailand [8], Brazil [9], Colombia [10], and China have underscored its potential for global dissemination [11, 12]. Clinical signs of PIVD mimic those of other vesicular diseases, complicating field diagnosis. Moreover, the virus exhibits high infectivity in feed [13, 14], and vectors such as mice and flies may facilitate its persistence in swine populations, emphasizing the need for prompt, accurate, and cost-effective diagnostic methods.

Current diagnostic approaches for SVA include serum neutralization assay [15], indirect enzyme-linked immunosorbent assay [16], competitive enzyme-linked immunosorbent assay [17], reverse transcription nested polymerase chain reaction [17], TaqMan-based quantitative reverse transcription PCR (qRT-PCR) [18], reverse transcription loop-mediated isothermal amplification [19], reverse transcription droplet digital PCR (RT-ddPCR) [20], and SYBR green I-based quantitative RT-PCR [21], each with inherent advantages and limitations. For instance, serum neutralization assay requires extended experimental periods; nested PCR is prone to cross-contamination; and techniques like qRT-PCR demand costly equipment. The CRISPR/Cas system, known for its precise sequence-specific recognition and cleavage abilities, has been employed extensively in genetic studies across various species due to its ability to target and editing specific genetic sequences [22–27]. Recent applications extend to viral detection, such as African swine fever virus (ASFV) and SARS-CoV-2 virus [28, 29]. FnCas12a, AsCas12a, and LbCas12a, three representative Type V Cas proteins, have been widely applied in pathogen detection due to their high cleavage activity and strict dependence on PAM sites [30, 31].

In this study, we utilize the LbCas12a protein, chosen for its robust cleavage activity and precise protospacer-adjacent motif (PAM) site dependence, to develop an ultra-sensitive, high-throughput, and fluorescence-based detection method for SVA. This method does not necessitate expensive reagents or complicated instrumentation. By leveraging the trans-cleavage activity of Cas12a combined with PCR amplification, we achieved a detection limit of one viral copy within three hours and 100% accuracy with simulated samples. This method promises significant advantages for large-scale disease surveillance and could play a pivotal role in controlling PIVD outbreaks.

## Results

### The CRISPR/Cas12a-based SVA fluorescent detection system and crRNAs design

**Fig. 1:**
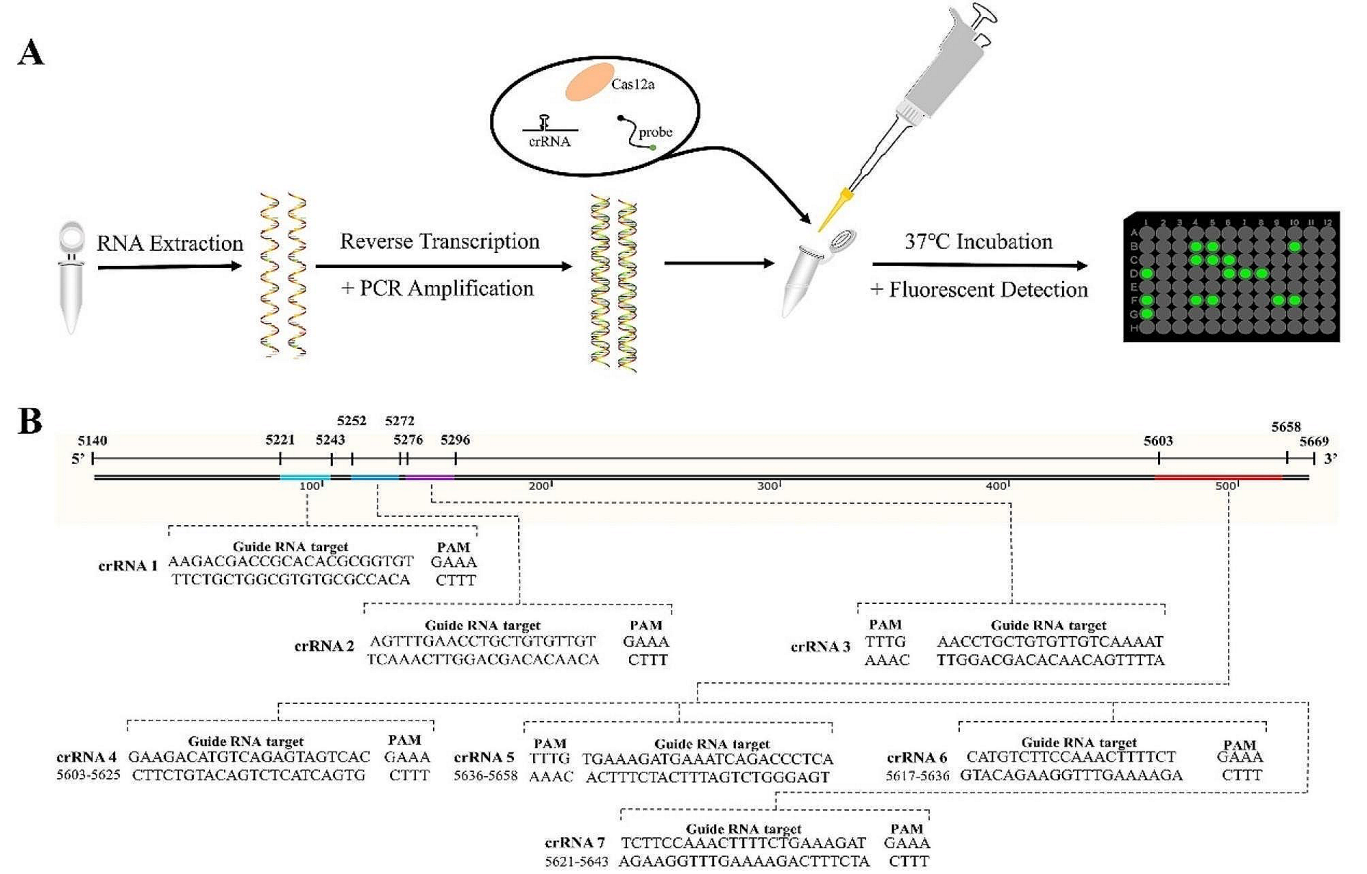
Schematic and target sites of crRNAs. (**A**) Overview of the CRISPR/Cas12a-based SVA fluorescent detection system. (**B**) Identification of 7 target sites of crRNAs within the SVA 3D gene

Traditional methods for SVA detection, such as RT-PCR and viral isolation, often fail to adequately balance sensitivity, cost, and equipment requirements. Advanced ultra-sensitive techniques such as RT-ddPCR require expensive equipment and specialized training. In contrast, the CRISPR/Cas12a-based SVA fluorescent detection system offers high sensitivity with minimal equipment requirements, involving four main steps: nucleic acid extraction, reverse transcription, pre-amplification, and fluorescence signal detection as depicted in Fig. 1A. Guided by crRNA, Cas12a specifically recognizes and binds to the target DNA sequence, triggering its non-specific trans-cleavage activity, which cleaves a fluorescent substrate, thus enabling rapid detection of SVA within three hours.

This study focuses on the 3D gene of Senecavirus A, a highly conserved gene, for primer and crRNA design. Twenty SVA genomes were retrieved from NCBI GenBank and aligned using SnapGene 4.2.4, leading to the creation of well-conserved primers and seven crRNAs, with the target sites illustrated in Fig. 1B. The sequences of the primers for the amplification of 3D gene are as follows: forward primer 5’-CCTCTGGCTACAATGCAAGGA-3’, and reverse primer 5’-CGGACCTTCTCTGAGGGT-3’.

### Trans-cleavage system optimization

**Fig. 2:**
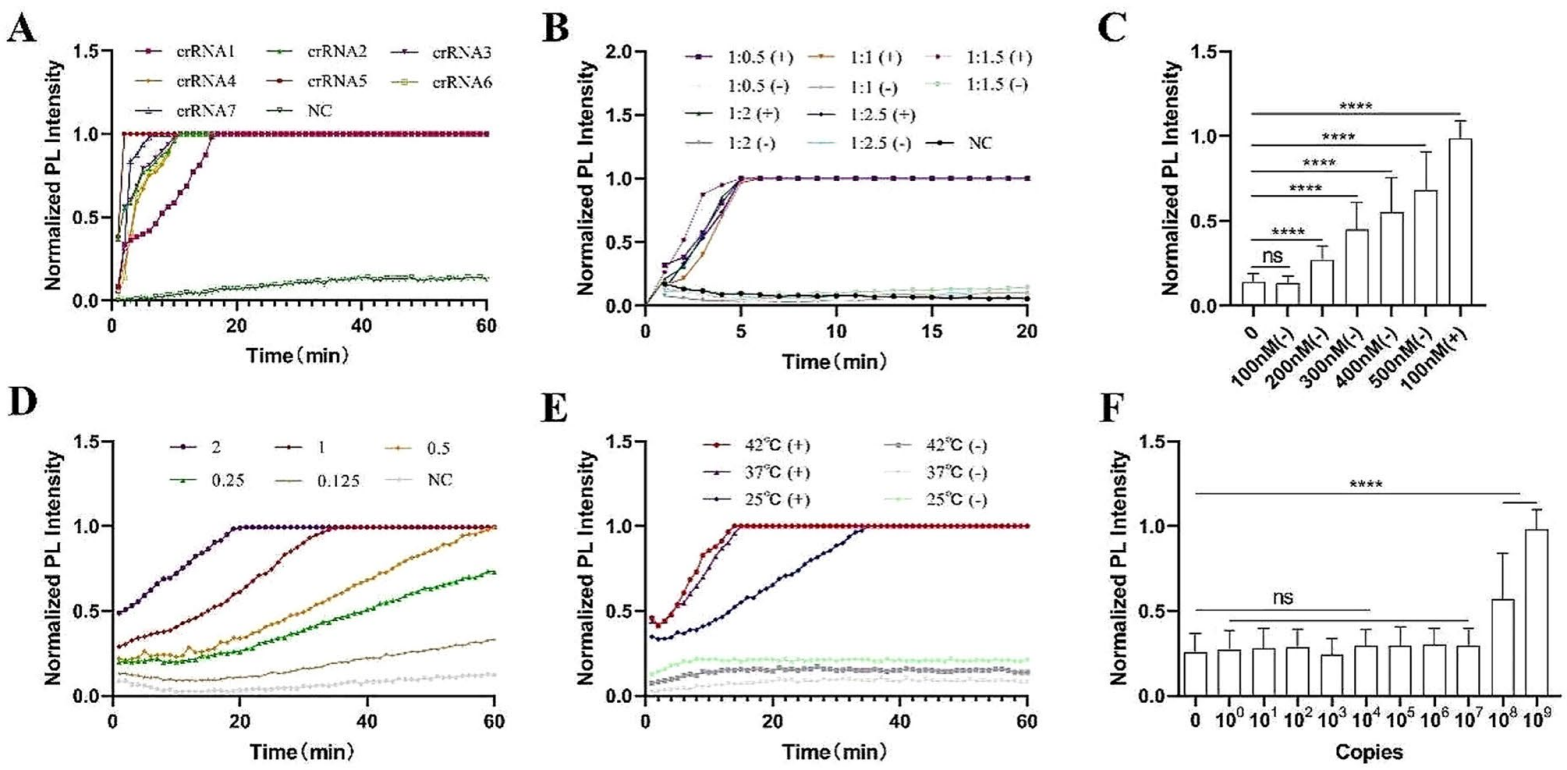
Optimization of the CRISPR/Cas12a-based SVA fluorescent detection system. (**A**) Screening of the most effective crRNAs. (**B**) Determination of the optimal Cas12a to crRNA ratio. (**C**) Determination of the optimal Cas12a concentration. (**D**) Identification of the optimal substrate (FAM-BHQ2 ssDNA reporter) concentration. (**E**) Optimization of the incubation temperature for Cas12a trans-cleavage activity. (**F**) Determination of the minimum template copies required for significant trans-cleavage activity. Nuclease-free water was used as a negative control (NC). 10^9^ copies/sample of SVA templates (pUC57-SVA-3D) was used to perform trans-cleavage system optimization. Bar graph data represent the mean ± standard deviation (SD) of three experimental replicates (****, *p* < 0.0001)

Enhancements to the CRISPR/Cas12a-based SVA detection system involved critical component tuning within a 100 µL reaction volume. Of the seven different crRNAs tested, crRNA5 emerged as the most efficient activator of trans-cleavage activity (Fig. 2A). Analysis of the Cas12a/crRNA interaction revealed that a 1:1.5 ratio yielded the steepest PL signal amplification curve (Fig. 2B). The concentration, Kcat, and Km of prepared LbCas12a protein were 0.41 mg/mL (20 µM), 4.03 ± 0.09 × 10^− 1^/sec, and 6.48 ± 0.38 × 10^− 7^/M (Table S1). The optimal concentration of Cas12a protein was found to be 100 nM, which minimized non-specific signals while maintaining robust trans-cleavage activity; higher concentrations led to an increase in non-specific signals and false positive results (Fig. 2C). The substrate concentration necessary for effective trans-cleavage activity was determined to be 10 nM, monitored using the Spark® multi-mode microplate reader (Fig. 2D). While changes in incubation temperature did not alter the final PL signal, they significantly influenced substrate conversion rates, with trans-cleavage requiring 34 min at 28℃ but only 12 to 13 min at 37℃ and 42℃, respectively (Fig. 2E). The minimum template concentration needed to activate significant trans-cleavage activity was determined to be 10^6^ copies/µL (Fig. 2F). The optimized reaction system thus comprises 100 nM purified Cas12a, 150 nM crRNA5, 10 nM substrate, 1× NEBuffer 2.1, 1 U/µL RNase inhibitor, the template (≥ 10^6^ copies/µL), and RNase-free water.

### Evaluation of specificity and sensitivity

**Fig. 3:**
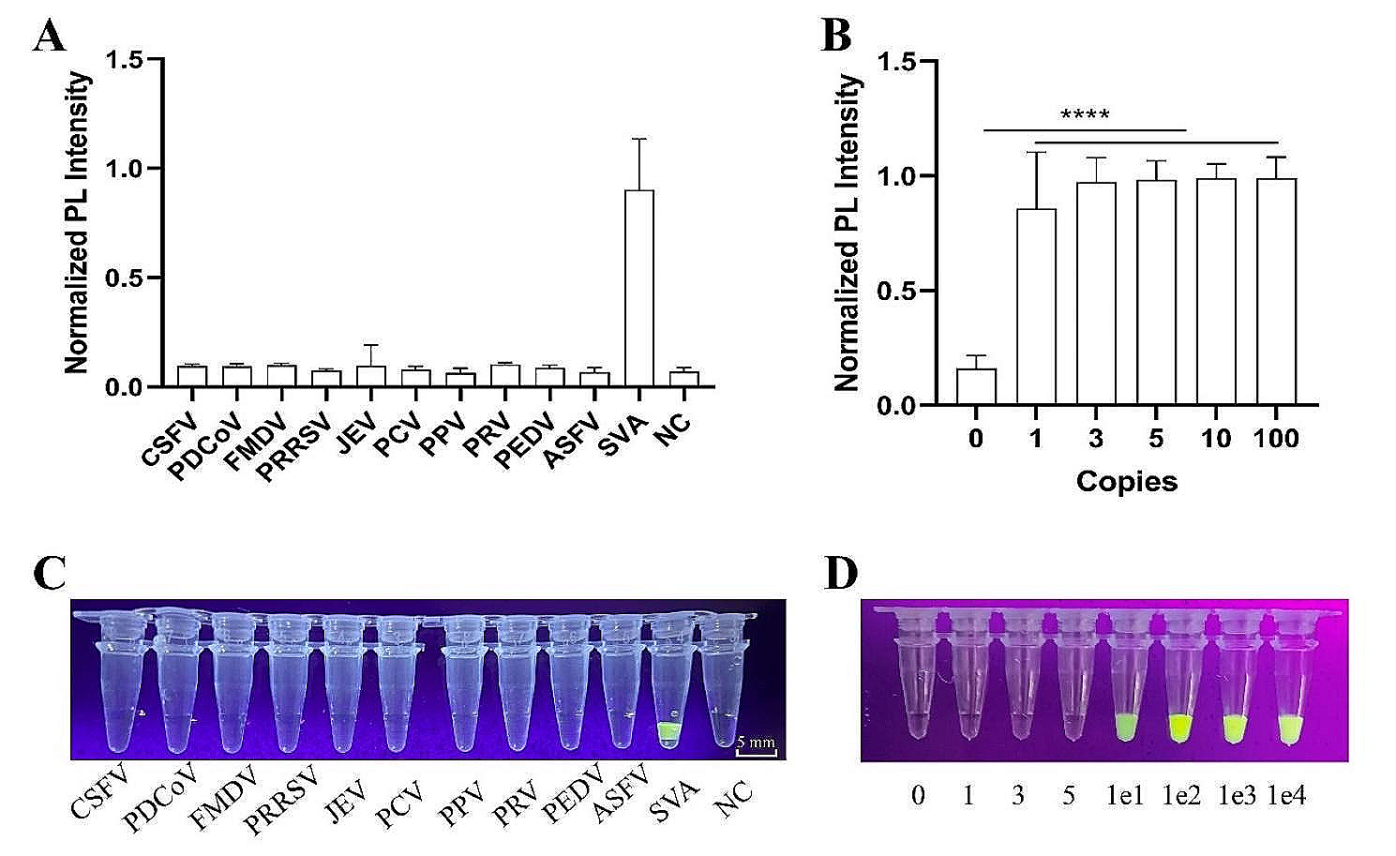
Specificity and sensitivity of the CRISPR-powered SVA detection method. (**A**) and (**C**): specificity of CRISPR-powered SVA detection method targeting 11 common swine viruses using MR or UV sensor system, respectively. (**B**) and (**D**): limit of detection of CRISPR-powered SVA detection method for gradient dilution of SVA templates (pUC57-SVA-3D) after PCR amplification. An aliquot containing nuclease-free water was used as a negative control (NC). Bar graph data represent the mean ± standard deviation (SD) of three experimental replicates (****, *p* < 0.0001)

The specificity of this method was assessed using 11 kinds of viruses of swine, including SVA, CSFV, PDCoV, FMDV, PRRSV, JEV, PCV, PPV, PRV, PEDV, and ASFV. In the MR sensor system, only SVA generated a robust PL signal, highlighting the method’s high specificity and utility for distinguishing SVA from other viruses of swine (Fig. 3A). The limit of detection (LOD) was established at 1 copy/reaction of targeted sequence (Fig. 3B). In the UV sensor system, non-target viruses did not activate Cas12a trans-cleavage activity; only SVA did (Fig. 3C). The LOD reached 10 copies/reaction of targeted sequence (Fig. 3D).

### Analysis of performance for simulated samples

**Fig. 4:**
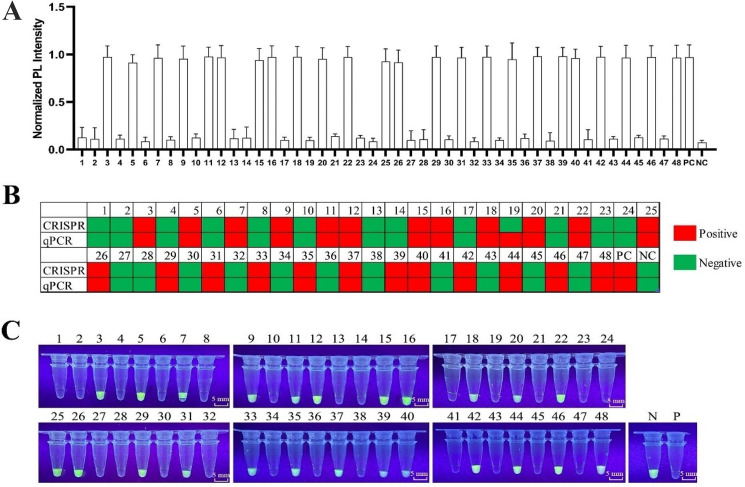
Detection performance of testing methods for 48 simulated samples. (**A**) Detection performance of the CRISPR-based SVA detection method using the MR sensor system. (**B**) Comparison of diagnostic results using the CRISPR-powered and qPCR-based SVA detection methods, with green indicating negative and red indicating positive results. (**C**) Detection performance of the CRISPR-based SVA detection method using the UV sensor system. An aliquot containing PCR products of SVA template or nuclease-free water was analyzed as positive (P) or negative (N), respectively

Among the 48 simulated samples, 24 samples were identified as positive by the CRISPR/Cas12a-based SVA detection method and 25 samples by the qPCR method (Fig. 4). A satisfactory overall concordance was achieved between the MR and UV sensor systems. As expected, samples not supplemented with SVA were diagnosed as negative (*n* = 24), while those supplemented were confirmed positive (*n* = 24) across the sensor systems (Fig. 4A and C). The qPCR results were consistent with the CRISPR method, except for one misdiagnosis in sample No.19, where CT values from three repeats were positive (Fig. 4B). Compared to qPCR, our method offers more precise detection of SVA.

## Discussion

Clinical manifestations of the PIVD closely mimic those of other vesicular diseases, such as foot-and-mouth disease and swine vesicular disease, complicating clinical differentiation. The World Organisation for Animal Health recommends several laboratory methods for SVA detection, including virus isolation, ELISA, RT-PCR, and RT-qPCR. Among these, RT-qPCR is the most prevalent but requires costly equipment and specialized personnel, restricting its use in remote or under-resourced laboratories. This highlights the necessity for innovative diagnostic techniques. Leveraging CRISPR/Cas12a, various diagnostic approaches for porcine viruses have been formulated [32]. In this study, we developed a novel method for Senecavirus A (SVA) detection, incorporating CRISPR/Cas12a technology, PCR, and fluorescence signal sensor systems. The method operates by the crRNA’s base pairing with the target sequence, activating Cas12a’s enzymatic activity to cleave a Fam-marked ssDNA and produce a fluorescent signal, thereby facilitating SVA detection.

Compared to traditional molecular diagnostics, the CRISPR/Cas12a-based SVA fluorescent detection system offers enhanced specificity and resolution. The CRISPR-Cas12a protein, derived from the type V-A CRISPR system, is an endonuclease that harbors a single RuvC catalytic domain and specifically recognizes a T-rich PAM site in the target DNA sequence [33]. This interaction allows for precise single-base discrimination by the crRNA-DNA template combination, preventing activation of the trans-cleavage activity in non-target scenarios, thus broadening Cas12a’s diagnostic application [34–36].

Furthermore, the CRISPR/Cas12a-based SVA detection method is not constrained by stringent ambient temperature requirements, with accurate results obtainable at incubation temperatures ranging from 28 °C to 42 °C. The cost per test is approximately $1.7. Although the method initially requires a minimum of 10^6^ copies/µL of the target sequence to activate trans-cleavage and produce a PL signal, pre-amplification strategies can address this limitation. The LOD for developed method is remarkably low, at one copy per reaction, significantly surpassing the sensitivities of qRT-PCR and traditional PCR [20]. The accuracy and stability of developed method were confirmed through the testing of simulated samples, underscoring its potential clinical utility for screening PIVD and mitigating its spread.

## Conclusion

We have successfully developed an ultra-sensitive, rapid, and high-specificity CRISPR-powered SVA detection method. This method incorporates the CRISPR/Cas12a system, DNA pre-amplification, and two distinct sensor systems. It enables flexible adjustments to the experimental setup in remote testing sites or well-equipped laboratories, thereby enhancing the control of SVA in the future.

## Methods

### Cas12a protein expression and purification

The LbCas12a protein, originating from *Lachnospiraceae bacterium*, was expressed using the previously established recombinant plasmid [33]. Expression commenced by transforming the pET28a-LbCas12a plasmid into *Escherichia coli BL21 (DE3)* cells (Tsingke, Beijing, China). Upon reaching the logarithmic phase during incubation at 37 °C in a shaker, 1 mM isopropyl-β-D-thiogalactoside (Sangon, Shanghai, China) was added to induce protein expression. After a further 5.5 h of incubation, cells were harvested by centrifugation at 6000 × g, washed thrice with PBS, and resuspended in lysis buffer (20 mM Na_3_PO_4_·12H_2_O, 20 mM NaCl, 20 mM imidazole). Cell lysis was performed using an ultrasonic disruptor, followed by centrifugation to remove cell debris. The clear supernatant was then filtered through a 0.22 μm filter and subjected to affinity chromatography using a His Trap FF column on an AKTA pure chromatography system (GE Healthcare, Little Chalfont, Buckinghamshire, UK). Cas12a was eluted using a gradient of imidazole and its presence was confirmed by SDS-PAGE assay. The purified protein was subsequently dialyzed in a solution containing 600 mM NaCl, 2 mM DTT, 50 mM Tris-HCl (pH 7.5), and 5% glycerol. Protein concentration was determined using a BCA kit (Smart-Lifesciences, Changzhou, Jiangsu, China) and stored at -80 °C until usage.

### Nucleic acid extraction, reverse transcription, and PCR

Nucleic acids were extracted using either the High Purity Total RNA Extraction Kit (Proteinssci, Shanghai, China) for RNA or the Viral DNA Kit (Omega Bio-Tek, Norcross, GA, USA) for DNA. Reverse transcription of RNA involved the EasyScript One-Step gDNA Removal and cDNA Synthesis SuperMix kit (TransGen, Beijing, China). The reaction setup included 7 µL of RNA sample, 10 µL of 2×ES Reaction Mix, 1 µL of Random Primer (0.5 µg/µL), 1 µL of EasyScript® RT/RI Enzyme Mix, and 1 µL of gDNA Remover, incubated at 42 °C for 30 min followed by inactivation at 85 °C for 5 s. PCR amplification utilized 2×Tsingke Master Mix (Tsingke, Beijing, China), with a reaction volume of 50 µL comprising 2 µL of template, 25 µL of 2× Master Mix, 2 µL each of 10 µM forward and reverse primers, and 19 µL of RNase-free water. The PCR protocol involved an initial denaturation at 95 °C for 5 min, followed by 35 cycles of denaturation at 95 °C for 30 s, annealing at 55 °C for 30 s, extension at 72 °C for 30 s, and a final extension at 72 °C for 10 min.

### **Enhancement of the CRISPR/Cas12a trans-cleavage efficiency**

The development of the CRISPR/Cas12a trans-cleavage efficiency encompasses several critical parameters: substrate concentration, the ratio of Cas12a to crRNA, Cas12a concentration, incubation temperature, and template concentration. This investigation utilized a 100 µL trans-cleavage reaction system comprising Cas12a, crRNA, template, RNase inhibitor, 1×NEBuffer™ 2.1 (New England Biolabs, Beverly, MA, USA), and RNase-free water. Fluorescent signals from 5-Carboxyfluorescein (Fam) were recorded every two minutes at 37℃ over a 60-minute period using the Spark^®^ multi-mode microplate reader (Tecan Trading, Shanghai, China). A molar gradient of 0.125 pmol (1.25 nM) to 2 pmol (20 nM) of the FAM-BHQ2 ssDNA reporter (5’-Fam-NNNNNNNNNNNN-BHQ1-3’) established optimal substrate concentrations for cleavage; a molar ratio gradient from 1:0.5 to 1:2.5 determined the optimal Cas12a/crRNA ratio; Cas12a concentrations ranged from 100 nM to 500 nM; temperatures of 25℃, 37℃, and 42℃ were tested for optimal incubation conditions; and target sequence copies from 10^0^ to 10^9^ gauged the activation threshold for significant photoluminescence (PL) intensity.

### Specificity and sensitivity of the CRISPR/Cas12a-Based SVA fluorescent detection method

The optimized conditions were applied to ascertain the specificity and sensitivity of this method. Furthermore, to enable use in poorly equipped laboratories at remote testing sites, an ultraviolet (UV) light transilluminator (Peiqing, Shanghai, China) was introduced to develop a colorimetry-based SVA CRISPR/Cas12a detection. The concentration of FAM-BHQ2 ssDNA reporter in a 20 µL of CRISPR/Cas12a reaction mixture was 1 µM. After incubating at 37 °C for 60 min, the reaction mixtures were exposed to a UV light transilluminator set at 146 mW/cm² to enable the detection of the fluorescent signal.

For ascertaining the specificity, the system was evaluated against a panel of viruses including foot-and-mouth disease virus (FMDV, O serotype), pseudorabies virus (PRV), Japanese encephalitis virus (JEV), porcine parvovirus (PPV), classical swine fever virus (CSFV), porcine reproductive and respiratory syndrome virus (PRRSV, North American prototype), porcine circovirus 2 (PCV2), porcine deltacoronavirus (PDCoV, China lineage), porcine epidemic diarrhea virus (PEDV, GII genotype), and African swine fever virus (ASFV, genotype II), with CCID_50_ values ranging from 1 × 10^4.5^ to 1 × 10^6.5^, provided by the China Veterinary Culture Collection Center (Beijing, China). The CRISPR/Cas12a trans-cleavage reaction and fluorescent signal capture, targeting these genomes, were performed to ascertain specificity, following reverse transcription and pre-amplification.

To ascertain the sensitivity of our detection system, the recombinant plasmid pUC57-SVA-3D of 10^0^ to 10^9^ copies of SVA templates underwent DNA extraction, reverse transcription, and pre-amplification. These amplified products were then utilized to determine the detection limits of our method, with each experiment conducted in triplicate.

### Detection of simulated samples

A mixture of various SVA-free tissues, including heart, liver, spleen, lung, kidney, tonsil, and lymph nodes, was sourced from a slaughterhouse in Nanjing. These samples were meticulously cut and ground to ensure homogeneity. Forty-eight samples were prepared, each containing 0.5 g of the tissue mixture and 1 mL of PBS. Half of these samples were spiked with 1 × 10^5^ CCID_50_ of SVA. After grinding and triple freeze-thaw cycles, the supernatants were collected via centrifugation at 13,000 × g for 10 min at 4 °C. These supernatants underwent nucleic acid extraction, reverse transcription, PCR, and CRISPR/Cas12a-based SVA detection. To evaluate diagnostic performance of our method, a previous SYBR Green I-based qRT-PCR method was adapted and compared using the Applied Biosystems StepOne™ Real-Time PCR System (Thermo Fisher Scientific, Waltham, MA, USA) and PerfectStart^®^ II Probe qPCR SuperMix (TransGen, Beijing, China) [21]. A sample was deemed positive if two or three of its replicates tested positive.

### Electronic supplementary material

Below is the link to the electronic supplementary material.

Supplementary Material 1

## Data Availability

All data generated or analyzed during this study are included in this article.
