# Supplementary material for: Development of a CRISPR/Cas12a-based fluorescent detection method of Senecavirus A
Source: BMC Vet Res. 2024 Jun 14;20:258. doi: 10.1186/s12917-024-04116-6 (PMC11179212; doi:10.1186/s12917-024-04116-6)
Supplement: Supplementary file 1 — Supplementary Material 1 [file 12917_2024_4116_MOESM1_ESM.docx]

Table S1 The Mie constant of LbCas12a

| Concentration (mg mL^-1^) | Molar concentration (μM) | Kcat (sec^-1^) | Km (M) | Kcat/Km (sec^-1^ M^-1^) |
| --- | --- | --- | --- | --- |
| 0.41 | 20.00 | 4.03 ± 0.09×10^-1^ | 6.48 ± 0.38×10^-7^ | 5.91±2.57×10^5^ |

Protein concentration was determined using a BCA kit (Smart-Lifesciences, Changzhou, Jiangsu, China). The optimal conditions determined in “Trans-cleavage System Optimization” were employed for testing the enzymatic activity of prepared LbCas12a protein in this study. 0.1 pmol to 10 pmol of pUC57-SVA-3D plasmids were used as activator.
